# Plasma Corin: A New Biochemical Marker for Polycystic Ovary Syndrome

**DOI:** 10.1007/s43032-024-01531-w

**Published:** 2024-04-26

**Authors:** Mohamed Abdel-moniem Ibrahem, Amira Saber Al-Karamany, Marwa M. Esawy, Amina Nagy Elasy

**Affiliations:** 1 Department of Obstetrics and Gynecology, Faculty of Medicine, Zagazig University, Zagazig, Egypt; 2 Department of Biochemistry and Molecular Biology, Faculty of Medicine, Zagazig University, Zagazig, Egypt; 3 Department of Clinical Pathology, Zagazig University, Zagazig, Egypt

**Keywords:** Biomarker, Corin, Polycystic ovary syndrome, Infertility

## Abstract

**Introduction:**

Polycystic ovary syndrome (PCOS) is a prevalent endocrine disorder. Atrial natriuretic peptide (ANP) is a risk factor for PCOS. Corin protein has an essential role in ANP synthesis. This study aimed to evaluate corin as a sensitive biomarker for PCOS.

**Materials and Methods:**

A case-control study was conducted with 70 PCOS patients and 70 healthy females. Plasma Corin levels were quantified using enzyme-linked immunosorbent assay.

**Results:**

The median plasma corin levels in PCOS patients and controls were 1785 and 822.5 pg/mL, respectively. Plasma corin levels were significantly elevated in PCOS patients than in the controls (*p* < 0.001). The optimal cut-off value was set at 1186 pg/mL. The sensitivity and specificity of Corin were 100% and 97.1%, respectively. Plasma corin levels were surrogate predictors for infertility in women with PCOS. It had an odds ratio of 5.9 (95% confidence interval: 1.1–32.7) (*p* = 0.04). Plasma corin levels were more highly detected in patients with PCOS than in the controls.

**Conclusion:**

Plasma corin level has reasonable diagnostic interpretation for PCOS. Corin appears as a worthy distinct predictor of infertility in PCOS women. Therefore, Corin may be a substantial biomarker for PCOS.

## Introduction

Polycystic ovary syndrome (PCOS) is a ubiquitous endocrine disorder that affects nearly 15% of women during their reproductive age. This prevalence was based on the Rotterdam criteria [1]. The main PCOS characteristics include menstrual or ovulatory irregularities, features of hyperandrogenism, and sonographic appearance of polycystic ovaries [2]. Common metabolic disturbances, such as obesity, dyslipidemia, and insulin resistance, have also been associated [3]. PCOS has a heterogeneous presentation and severity that might change over time [4].

PCOS pathogenesis is multifactorial. Numerous genetic, intrauterine, and environmental mechanisms have been implicated in this process. Obesity is a major postnatal environmental factor [5]. Adipose tissue dysfunction plays a role in PCOS aggravation and the development of its associated metabolic disorders [6], particularly the subcutaneous adipose tissues, which participate in extra ovarian sources of estrogen via androgen aromatization [7].

Corin is a transmembrane serine protease (EC 3.4.21) that is mainly involved in atrial natriuretic peptide (ANP) and B-type natriuretic peptide synthesis [8]. In humans, the cytogenetic location of the corin gene is 4p-12. The highest expression of Corin is notable in the cardiac muscles [9]. The regulation of Corin expression is maintained through various cellular processes, such as transcription, translation, and ectodomain shedding [10]. Soluble Corin protein seems to be a precise biomarker for cardiovascular diseases, including risk assessment and prognosis [11]. Furthermore, Corin has been reported to be a risk factor for both dyslipidemia and endothelial dysfunction [12, 13].

Recently research has been conducted to evaluate the correlation between ANP and various metabolic disorders including PCOS [1, 14]. Since corin is considered an essential protein in the pathway of ANP synthesis, this study advocates that PCOS adipose tissue and metabolic changes could be manifested by abnormal corin levels. Thus, our study aimed to elucidate the role of Corin in PCOS by measuring its plasma levels in PCOS patients and healthy controls. And to evaluate corin as a biomarker for PCOS and to examine its association with PCOS characteristics.

## Materials and Methods

### Study Design & Sitting

**Fig. 1:**
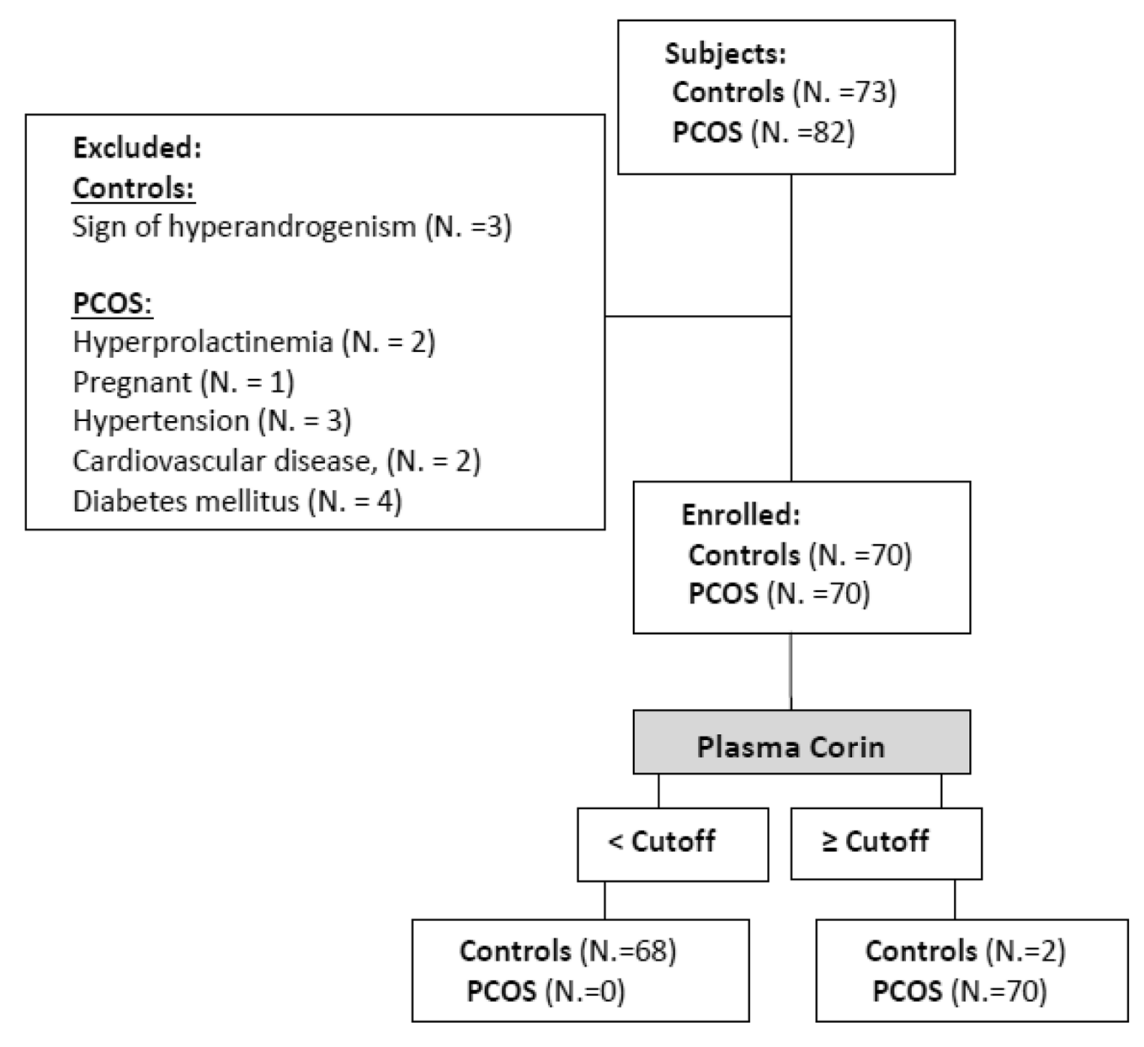
Flowchart of this study

A case-control study was conducted on 70 PCOS patients between February and May 2023. Seventy healthy females were enrolled as the control group (Fig. 1). Both groups were matched for their demographic characterizations. The participants were recruited from the Obstetrics and Gynecology Clinic, and laboratory tests were performed at the Clinical Pathology Department, Faculty of Human Medicine, Zagazig University. Informed written consent was obtained from all the patients. Inclusion criteria: The selection criteria for eligibility ranged from 18 to 40 years. The participants of the control group had regular menstrual cycles and no signs of hyperandrogenism. The diagnosis of PCOs was based on the Rotterdam Consensus Criteria [15]. Exclusion Criteria: Women who had causes of oligomenorrhea or hyperandrogenism other than PCOS (such as Cushing’s syndrome, hyperprolactinemia, and congenital adrenal hyperplasia) were excluded from this study. Pregnancy and other comorbidities (such as hypertension, cardiovascular disease, or diabetes mellitus) were excluded from the study. Additionally, women who used oral contraceptives, hormonal therapy, or anti-lipidemic drugs were excluded.

Sample Size: A total sample of 140 subjects of equal group sizes was calculated to achieve a 95% confidence interval and 80% power. The difference in mean values and the pooled standard deviation were derived from a previous study addressing corin in obesity [13].

### Definitions

Hypertension was defined when blood pressure measures ≥ 140/ 90 mmHg, or the current use of antihypertensive drugs. Diabetes was defined as a previous diagnosis of diabetes, use of antidiabetic drugs, or a level of fasting glucose at or exceeds 126 mg/dL. Cardiovascular disease was defined as prior angina pectoris, myocardial infarction, transient ischemic attack, or stroke. In this study, infertility was defined as PCOS-induced infertility, and other causes of infertility were ruled out.

### Patients Recruitment

PCOS patients were screened for inclusion and exclusion criteria. The patients were recruited continuously until the desired sample size was achieved. Controls were randomly recruited from among women who consulted for contraception. The same exclusion criteria were applied to the controls. This study used an age- and BMI-matched design. The controls were matched for age (± 5 years) and BMI (± 3 kg/m2) of PCOS patients.

### Study Tools

The study participants were subjected to comprehensive history taking, clinical examination, gynecological examinations, and pelvic ultrasound. Laboratory hormonal, lipid, and glucose metabolism profile assessments were performed. Hormonal profiles included luteinizing hormone (LH), follicle-stimulating hormone (FSH), total testosterone, and dehydroepiandrosterone sulfate (DHEA-S). The lipid profile included triglycerides, total cholesterol, high-density lipoprotein cholesterol (HDL-C), and low-density lipoprotein cholesterol (LDL-C). The glucose metabolism profile included fasting glucose and fasting insulin measurements as well as a homeostasis model assessment for insulin resistance index (HOMA-IR) calculation.

Serum samples were measured on the 2nd or 3rd day of spontaneous or induced menses for hormonal profile assessment. After sample collection, the enrolled subjects were instructed to fast from midnight onwards. The next day, at 8 a.m., serum and plasma samples were collected to assess the blood glucose profile and Corin, respectively. At noon, another serum sample was obtained to evaluate the lipid profile.

### Sample Collection and Preparation

Peripheral blood samples were obtained by venipuncture into BD Vacutainer ® Plus plastic serum tubes and EDTA tubes for serum and plasma separation, respectively (Becton, Dickinson and Company, Franklin Lakes, NJ, USA). After sample collection, the serum tubes were allowed to clot at room temperature for 30 min. Subsequently, they were centrifuged, and EDTA types were immediately centrifuged. The centrifugation force used to separate the serum and plasma was 1200 g for 10 min. All laboratory tests were performed immediately, except for corn measurements. Plasma aliquots (0.5 mL) were stored at – 80 ºC for corin assessment.

### Methods

A Cobas 8000 Modular Analyzer was used to perform the laboratory tests (Roche Diagnostics, Mannheim, Germany) [16]. The assessment of both lipid profile and glucose levels was conducted by using the c-702 module, whereas the e-602 module was utilized for hormone assessment. Total blood levels of cholesterol, triglyceride, and glucose were scaled using standard enzymatic colorimetric methods [17]. HDL-C was quantified using a third-generation direct method [18]. LDL-C levels were measured using the second-generation direct method [19]. Hormones were measured using the electrochemiluminescence method presented in commercial Elecsys kits [20]. All reagents, calibrators, and quality controls were purchased from Roche Diagnostics (Mannheim, Germany). Parallel quality controls were used for each run.

The Corin levels were measured by the enzyme-linked immunosorbent assay (ELISA) [10, 21]. Human CORIN ELISA Kit (MyBioSource, San Diego, CA, USA [catalog number: MBS824802] was used in this study. This was a quantitative sandwich enzyme immunoassay technique. The steps were carried out following the recommended steps of the manufacturer. Briefly, 100 µL of the standards and samples were added, and the plate was incubated for 90 min at room temperature. The plate was washed 3 times,

Next, 100 µL of biotin-labeled detection antibody working solution was added and incubated at 37 °C for 60 min. After three wash cycles, a 100 µL streptavidin-HRP working solution was added. The plates were then incubated for 45 min at 37 °C. After 5 wash cycles, 100 µL of TMB substrate solution was added and incubated at 37 ºC in the dark for 30 min. The reaction was stopped by adding 100 µL stop solution. Optical density (OD) was measured at 450 nm using a Sunrise™ absorbance reader (Tecan Trading AG, Männedorf, Switzerland). A paradigmatic curve was created from the relative OD of each standard solution and its respective concentrations. Each sample was analyzed twice. The accepted coefficient of variation (CV) between the two measurements was < 10%. For each sample, the corin concentration was calibrated as the average of two measurements. The corin results are expressed as (pg/mL).

This ELISA had a measurement range extending from 62.5 to 4000 pg/ml, which was sufficient to cover the expected levels of Corin. The minimum detectable dose of Corin is < 10 pg/mL. The precision of the ELISA was assessed using three samples with different corin concentrations. These samples were measured 20 times using one plate to estimate the intra-assay precision and were measured 20 times using assays to estimate the inter-assay precision. The Average intra-assay CV was 4.1%, whereas the inter-assay CV was 7.2%.

### Calculations

Body mass index (BMI) was measured as weight (kg)/height (meters). HOMA-IR was estimated using the following formula for fasting values: [insulin level (uIU/ml) × glucose level (mmol/L)]/22.5 [22].

### Statistical Analysis

The Mann-Whitney U test was used to perform data comparisons, and the chi-square test was used when appropriate. Analysis of covariance was performed using ANCOVA after logarithmic transformation of the data. The receiver operating characteristic (ROC) curve and its analysis were used to determine the diagnostic accuracy of Corin in PCOS. The correlation between Corin and PCOS characteristics was assessed using Spearman’s correlation test. A binary logistic regression analysis was used to clarify the predictive role of Corin. All statistical tests were performed using the SPSS 17.0 program (Chicago, IL, USA). Statistical significance was set at *P* < 0.05.

### Ethical Approval

The Institutional Review Board of the Faculty of Human Medicine at Zagazig University approved this study (IRB # 10,479).

## Results

In total, 140 Egyptian patients (70 PCOS patients and 70 healthy controls) were enrolled in this study. The clinical, biochemical, and hormonal characteristics of the participants are presented in Table 1. There was no statistically significant difference between the patients and controls in terms of age (*P* = 0.21). BMI showed borderline significance between patients and controls (*p* = 0.07). Compared to controls, patients had significantly higher HOMA-IR, triglyceride, total cholesterol, LDL-C, LH, LH/FSH ratio, and DHEA-S. In contrast, the HDL-C levels were significantly reduced (*p* < 0.05). However, the fasting glucose, fasting insulin, FSH, and total testosterone levels were not significantly different from those of the controls (*p* > 0.05).

**Table 1:** Subjects characteristics

Parameter	PCOS (No. = 70)	Control (No. = 70)	*p*
Age (years)	26 [20–37]	25 [19–32]	0.21
Infertility	52 (69.7)	---	
Oligomenorrhea	61 (87.1)	---	
BMI (Kg/m2)	27.4 [22.1–33.4]	25.9 [22.5–30.5]	0.07
Fasting glucose (mg/dL)	86.5 [70–140]	80.5 [70–110]	0.11
Fasting Insulin (µIU/mL)	5.5 [3.5–10.2]	5.2 [3.2–9.2]	0.23
HOMA-IR	1.2 [0.64–2.59]	1.0 [0.6–2.5]	0.041*
Total cholesterol (mg/dL)	126.3 [94.5-224.2]	117.4 [90.5-195.6]	0.01*
Triglyceride (mg/dL)	82.3 [60.4-147.5]	76.8 [49.7–112]	0.046*
HDL-C (mg/dL)	44.3 [32-62.2]	45 [34–60]	0.029*
LDL -C(mg/dL)	68.7 [21.4-146.3]	58.5 [43.1-130.9]	0.042*
FSH (mIU/mL)	5.2 [2.5–10.9]	5.9 [3.4–7.8]	0.06
LH (mIU/mL)	7.5 [5.1–19.5]	5.4 [2.5–12]	< 0.001*
LH/FSH ratio	1.8 [0.6–4.7]	1.0 [0.4–2.2]	< 0.001*
Total testosterone (ng/mL)	0.2 [0.1–1.4]	0.2 [0.08–0.39]	0.06
DHEA-S (µg/dL)	205.5 [99–412]	177.5 [95.0-324]	0.005*
Corin (pg/mL)	1785 [1325–2600]	822.5 [658–1952]	< 0.001*

No.: Number of subjects; BMI: Body mass index; HOMA-IR: Homeostatic model assessment of insulin resistance; HDL-C: High-density lipoprotein cholesterol; LDL-C: Low-density lipoprotein cholesterol; LH: Luteinizing hormone; FSH: Follicle-stimulating hormone; DHEA-S: Dehydroepiandrosterone sulfate

Data are presented as No. (%) or median [Range]

* Significant

**Fig. 2:**
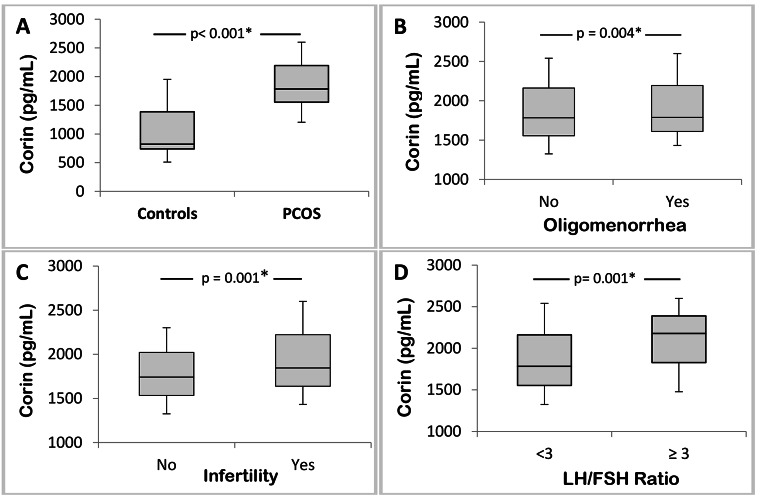
Box and whisker plot of Corin levels for (**A**) the study groups and (**B-D**) different subgroups of PCOS patients

The median plasma corin level was significantly detected in PCOS patients than in controls (1785 and 822.5 pg/mL, respectively) (*p* < 0.001) (Fig. 2A). ANCOVA test showed a statistically significant difference in plasma corin levels between PCOS patients and controls when adjusted for BMI (*p* < 0.001). Eighty-four of the variances in plasma corin levels were explained by PCOS after adjusting for BMI status.

Plasma corin levels were significantly higher in PCOS patients with oligomenorrhea than in those with regular menstrual cycles (*p* = 0.004). PCOS patients with infertility demonstrate significantly higher plasma corin levels than those with normal fertility (*P* = 0.001). Statistically significant increases were found in PCOS patients with an LH/FSH ratio equal to or higher than three (*p* = 0.001) (Fig. 2).

**Fig. 3:**
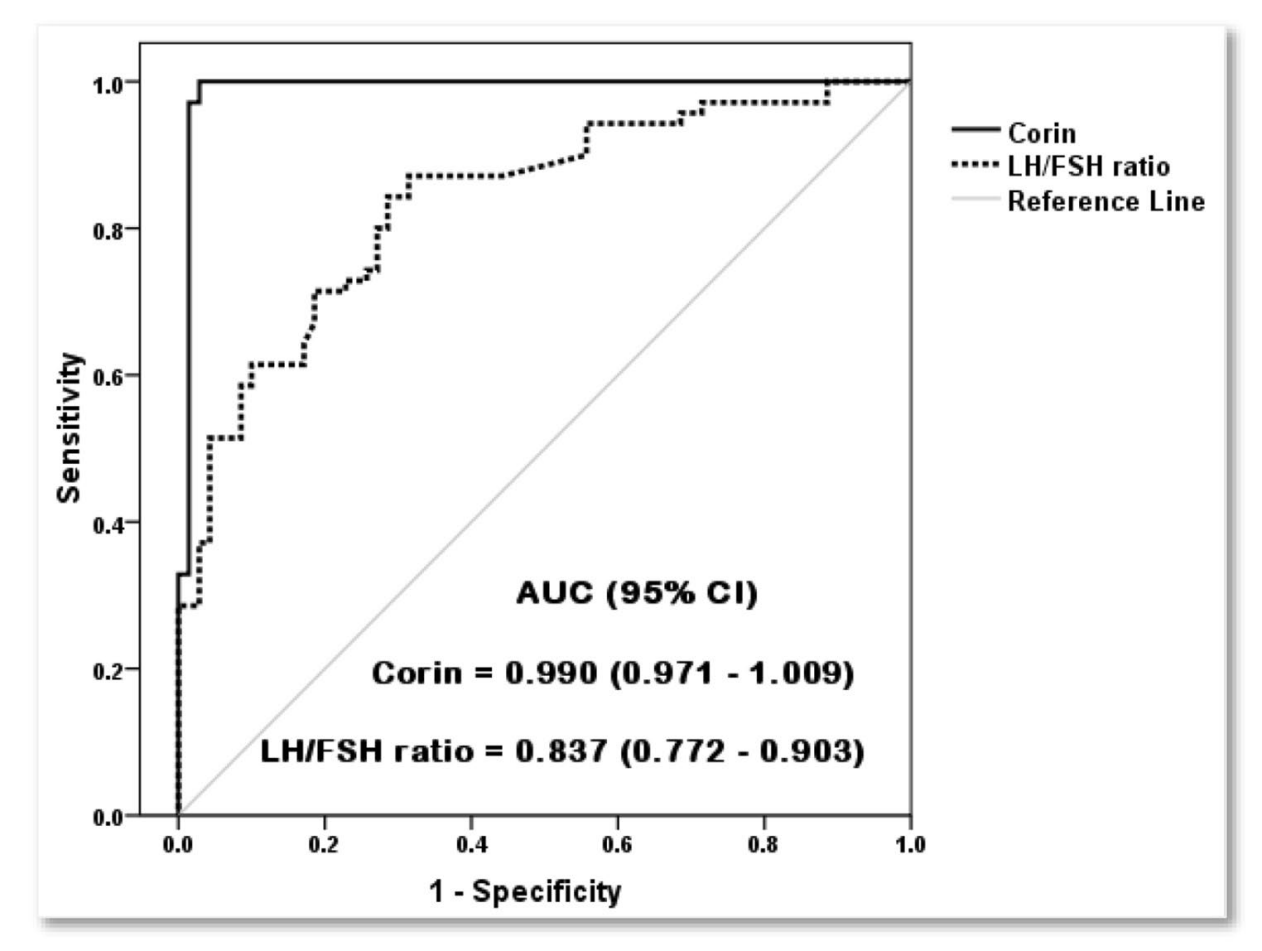
ROC curve for corin and LH/FSH ratio in PCOS prediction AUC: Area under curve; CI: Confidence interval

ROC curve analysis was used to assess the performance of Corin. The area under the ROC curve for Corin was 0.990 (Fig. 3). The optimal cutoff value was set at 1186 pg/mL. The sensitivity of Corin was 100%, while the specificity, positive predictability, and negative predictability were 97.1%, 97.2%, and 100%, respectively. The diagnostic accuracy of plasma corin level was 98.6%. The LH/FSH ratio, at a cut-off of 1.12, showed 84.3% sensitivity, 70% specificity, 73.8% positive predictability, 81.7% negative predictability, and 77.1% diagnostic accuracy in the detection of PCOS. The role of Corin as a potential predictor of PCOS was evaluated using Logistic Regression Analysis. Plasma corin showed an odds ratio of 1.1 (95% confidence interval: 1-1.2) (*p* < 0.0001).

In the study cohort, plasma corin levels correlated with hormonal, lipid, and glucose metabolism profiles. For PCOS patients, correlation analysis showed a significant direct correlation between plasma corin and BMI, HOMA-IR, total cholesterol, LDL-C, LH/FSH ratio, and DHEA-S. Plasma corin was positively correlated with BMI and HOMA-IR in healthy controls (Table 2).

**Table 2:** Spearman’s correlation between corin levels and PCOS characteristics

Parameter	PCOS (No. = 70)		Controls (No. = 70)	
	r_s_	*p*	r_s_	*p*
Age	0.13	0.27	0.02	0.91
BMI	0.27	0.02*	0.30	0.013*
Fasting glucose	0.19	0.11	0.19	0.11
Fasting Insulin	0.23	0.06	0.23	0.06
HOMA-IR	0.29	0.014*	0.24	0.043*
Total cholesterol	0.26	0.03*	0.11	0.36
Triglyceride	0.01	0.93	-0.13	0.28
HDL-C	0.01	0.99	0.23	0.06
LDL -C	0.30	0.01*	0.03	0.83
FSH	-0.13	0.30	0.06	0.62
LH	0.13	0.28	0.22	0.07
LH/FSH ratio	0.24	0.04*	0.13	0.30
Total testosterone	0.21	0.08	0.07	0.59
DHEA-S	0.23	0.04*	0.14	0.25

rs: Spearman’s correlation coefficient; BMI: Body mass index; HOMA-IR: Homeostatic model assessment of insulin resistance; HDL-C: High-density lipoprotein cholesterol; LDL-C: Low-density lipoprotein cholesterol; LH: Luteinizing hormone; FSH: Follicle-stimulating hormone; DHEA-S: Dehydroepiandrosterone sulfate

* Significant

The key role of Corin as a potential predictor of infertility in PCOS patients was assessed using Logistic Regression Analysis. PCOS patients were classified into two groups based on the median corin level. Plasma Corin showed an odds ratio of 9.3 (a 95% confidence interval: 2.0–44.70) (*p* = 0.005). On multivariate analysis, the plasma corin level was a valuable independent predictor of infertility in PCOS patients. Plasma Corin had an adjusted odds ratio of 5.9 (a 95% confidence interval: 1.1–32.7) (*p* = 0.04). Plasma Corin was adjusted to the BMI, LH/FSF ratio, and HOMA-IR (Table 3).

**Table 3:** Univariate and multivariate logistic regression analysis for infertility predictors in PCOS patients

Parameters	Univariate logistic regression analysis		Multivariate logistic regression analysis	
	OR (95% CI)	*p*	OR (95% CI)	*p*
Corin	9.3 (2.0-44.7)	0.005*	5.9 (1.1–32.7)	0.04*
BMI	1.2 (1-1.5)	0.045*	1.2 (0.9–1.5)	0.16
LH/FSH ratio	2.9 (1.2-7.0)	0.019*	3.1 (1.0-9.9)	0.06
HOMA-IR	5.7 (0.8–40.4)	0.08	---	---

OR: Odds ratio, CI: Confidence interval; BMI: Body mass index; HOMA-IR: Homeostatic model assessment of insulin resistance; LH: Luteinizing hormone; FSH: Follicle-stimulating hormone

Multivariate model included corin, BMI, LH/FSF ratio, and HOMA-IR

* Significant

## Discussion

In Egypt, PCOS is considered a major female health problem [23]. However, the pathological mechanisms underlying PCOS are complex and not completely understood. The PCOS-associated adipose tissue metabolic dysfunction has figured out the important role of adipose tissue-regulating factors in its pathogenesis. The reduction of ANP levels in PCOS patients contributes as a common etiological factor [1, 24]. Corin explains new insights into the natriuretic peptide system. Conversely, the role of Corin in various physiological and pathological processes remains unclear. The role of Corin in PCOS pathogenesis has not been thoroughly evaluated. The diversity of the clinical presentations of women with PCOs hinders early recognition and accurate diagnosis. Ultimately, this will delay the implementation of lifestyle modification strategies and the management of patients. Therefore, studying the potential evidence-based preclinical biomarkers such as Corin will help the early prediction of PCOs associated risk. Additionally, it represents a significant endeavor toward successful diagnosis and management.

Corin is a transmembrane protease that is exposed to proteolytic shedding, similar to many membrane-bound proteins. Soluble Corin is detected in circulation [9]. The circulating form of Corin showed the same activity as ANP activation [25]. Therefore, soluble corin has been hypothesized to be associated with PCOS. Thus, a case-control study was conducted to analyze the role of plasma corin in PCOS.

This study evaluated the clinical, biochemical, and hormonal variables of PCOS patients compared to the healthy cohorts. Regarding the clinical features of the patients, this study found that 87% complained of oligomenorrhea, and about 70% had a history of infertility. Menstrual irregularity is the most prevalent manifestation of PCOS, affecting nearly 80% of patients are affected [26]. An infertility prevalence of 70–80% has been reported in PCOS [27].

Regarding biochemical variables, the current study evaluated the differences between cases and controls in glucose metabolism and lipid profiles. PCOS patients had elevated HOMA-IR values, but fasting blood glucose and fasting insulin levels were not different significantly from those of controls. Our results are in line with those of Wageh et al. [24]. Fasting glucose, fasting insulin, and HOMA-IR levels are importantly higher in patients with PCOS [28]. In contrast, Lauria et al. [1] found an unnoticeable difference between patients with PCOS and controls. This discrepancy in results may be associated with the heterogeneous presentation, severity, and phenotypes of the PCOS patients included in each study.

Dyslipidaemia is a well-recognized metabolic change in PCOS. A dyslipidemia prevalence of 70% has been established in PCOS [34]. Regarding the lipid profile, PCOS patients showed incredibly higher values of triglycerides, total cholesterol, and LDL-C when compared to healthy cohorts. However, HDL-C levels were significantly lower than those in the controls. This dyslipidemia pattern is often observed in PCOS [29].

Hormonal profiles were assessed in both study groups. Compared to controls, PCOS patients had higher LH and LH/FSH ratios. However, FSH levels were comparable between patients and controls. These abnormal gonadotropin secretions have been encountered as common endocrine abnormalities in PCOS [30]. Elevated androgen production has been reported in PCOS patients. The current study showed that PCOS patients had parallel increased DHEA-S levels compared to controls, but their total testosterone levels were comparable to those of the controls. These results agree with those of Arpaci et al. [31].

Peleg et al. [32] revealed that ELISA is a suitable method for corin quantification. This study used a commercial ELISA to measure plasma corin levels in all study subjects. Soluble corin stability in plasma samples was previously reported [33].

The detected plasma corin level in the control group was comparable to the one that was previously stated in healthy Egyptian individuals [34]. Plasma corin levels were detected significantly higher in PCOS patients than in controls. Similarly, plasma corin levels were elevated in PCOS patients with oligomenorrhea and those who had an infertility history. The plasma corn’s ability to distinguish between PCOS patients and healthy control was appreciated, and it showed acceptable performance criteria. These findings suggest that Corin may help improve PCOS diagnosis in addition to the Rotterdam criteria. Also, Corin could be a practical test for PCOS screening.

Plasma Corin was directly correlated with BMI, HOMA-IR, total cholesterol, and LDL–C. Peng et al. [13] stated that Corin was significantly correlated with BMI, total cholesterol, and LDL-C. The correlation between the LH/FSH ratio and DHEA-S was significant. LH-FSH ratio contributed as a diagnostic tool for PCOS [35]. Corin showed a positive correlation with the LH/FSH ratio, but it had higher specificity and sensitivity for PCOS prediction.

PCOS infertility is mainly attributed to anovulation, but its underlying mechanism is still uncertain [36]. Corin appeared to be a significant independent predictor of infertility in PCOS patients. High Corin contributed to PCOS infertility, not to obesity correlation. The probability of infertility was greater (5.9-fold) in PCOS patients with high Corin levels compared to those who had low Corin levels. Beyond the well-known links between PCOS, obesity, and metabolic syndrome, gonadal functions, and insulin sensitivity exhibit complex relationships. Important pathophysiological connections between metabolic syndrome and infertility may be explained by standard types of dysfunctional adiposity and altered adipokine production [37].

Circulating Corin was assessed in many diseases. Decreased corin levels were observed in osteoporosis [38]. But increased levels were detected in preeclampsia [39], hypertension [40], obesity [13], and hyperglycemia. However, the mechanisms of these changes in different diseases are still uncertain. The altered Corin reflected a new mechanism related to its associated diseases. Moreover, ANP regulates oocyte maturation and ovarian steroidogenesis. The ANP role in PCOS was suggested, and the associated ovulatory dysfunction contributed to lower ovarian and plasma ANP levels [1] Nevertheless, the growing evidence suggests that a reduced ANA level could predispose to insulin resistance. A low activity of natriuretic peptide activates the renin-angiotensin system. Consequently, it initiates the process of insulin resistance through suppression of intracellular insulin signaling, exaggerates oxidative stress, and inflammation, and decreases blood supply to the skeletal muscle and pancreas [24]. The current study revealed that Corin levels were increased in PCOS and were directly associated with the Rotterdam criteria. Therefore, as a primary activator of ANP, Corin could have a substantial role in PCOS pathology. Also, Corin may contribute to PCOS-associated insulin resistance and metabolic disturbances.

Several limitations were acknowledged. The sample size was relatively small which hinders the possibility of sub-classify the patients into different PCOS phenotypes. The PCOS diagnosis was performed according to Rotterdam diagnostic criteria only. The lack of Corin activity assessment in the studied subjects was another limitation. Further studies on many participants are recommended to confirm the correlation with PCOS. These studies are required to establish the Corin protein as a biomarker for PCOS. Also, studies are needed to explore PCOS pathogenesis since this can be a step in the development of new therapeutic modalities.

## Conclusion

Plasma corin showed increased levels among PCOS patients in comparison with controls. Plasma Corin has acceptable diagnostic performance criteria for PCOS. Corin seems to be a reliable independent predictor of infertility in PCOS patients and could be adopted as a biomarker for PCOS.

## Abbreviations

ANP: Atrial natriuretic peptide
BMI: Body mass index
DHEA-S: Dehydroepiandrosterone sulfate
FSH: Follicle-stimulating hormone
HDL-C: High-density lipoprotein cholesterol
HOMA-IR: Homeostasis model assessment for insulin resistance index
LDL-C: Low-density lipoprotein cholesterol
LH: Luteinizing hormone
PCOS: Polycystic ovary syndrome
ROC: Receiver operating characteristic
